# *Candida guilliermondii* as an agent of postpartum subacute mastitis in Rio de Janeiro, Brazil: Case report

**DOI:** 10.3389/fmicb.2022.964685

**Published:** 2022-09-23

**Authors:** Tatiane Nobre Pinto, Alana Kohn, Gisela Lara da Costa, Laura M. A. Oliveira, Tatiana C. A. Pinto, Manoel M. E. Oliveira

**Affiliations:** ^1^Instituto de Microbiologia Paulo de Góes, Universidade Federal do Rio de Janeiro, Rio de Janeiro, Brazil; ^2^Instituto Fernandes Figueira, Fundação Oswaldo Cruz, Rio de Janeiro, Brazil; ^3^Instituto Oswaldo Cruz, Fundação Oswaldo Cruz, Rio de Janeiro, Brazil

**Keywords:** candidiasis, mastitis, breastfeeding, yeasts, *Candida guilliermondii*, mammary infections

## Abstract

*Candida* spp. can cause mild-to-severe human infections. Certain species have been described as the etiologic agent of human mastitis, inflammation of the breast tissue. Mastitis affects millions of lactating women and can be a source of disease transmission to the infant. In this work, we report the detection of the unusual etiologic agent of human mastitis, *Candida guilliermondii*, isolated from the milk of a puerperal woman with subacute mastitis in Rio de Janeiro, Brazil. Species identification was performed by MALDI-TOF MS and genetic sequencing. The patient had a full recovery after antifungal therapy.

## Introduction

*Candida* spp. are commensals of the skin, mouth, and gastrointestinal tract (Wang et al., 2013). Overgrowth and spread of this yeast are generally impaired by coexisting members of the microbiome, by epithelial barriers, and by host immune defenses (Xu and Dongari-Bagtzoglou, 2015).

However, *Candida* spp. can transition to pathogenic status, being able to cause a wide spectrum of clinical conditions, ranging from superficial infections to life-threatening systemic diseases (Xu and Dongari-Bagtzoglou, 2015; Bradford and Ravel, 2017).

Previous studies report vaginal yeast infections at birth, the use of antibiotic during labor or postpartum, and the use of bottles and pacifiers as risk factors for developing mammary infections by *Candida* species (Morrill et al., 2005; Wiener, 2006; Hanna, 2011).

Mastitis is characterized as an inflammation of the breast and is more common in women who are breastfeeding. The symptoms of mastitis are mainly swelling, pain and redness in the breasts, fever, tiredness, and the presence of discharge. Complications of mastitis include the development of abscesses, interruption of breastfeeding, and an increased risk of neonatal infections (Espínola-Docioa et al., 2016; Angelopoulou et al., 2018; Boakes et al., 2018; Moubareck, 2021). The prevalence of mastitis in lactating women ranges from 2.5% to 40%, according to the geographical region (Wilson et al., 2020). In Brazil, studies on this clinical condition are still scarce. Menezes et al. (2004) reported the occurrence of *Candida guilliermondii* in 7.7% of nipple fissures samples recovered from lactating women assisted at the Human Milk Bank of the Assis Chateaubriand Maternity School of the Federal University of Ceará, Brazil.

*Candida guilliermondii* complex is a heterogeneous taxonomic group composed of several species that are morphologically indistinguishable, such as *C. guilliermondii, Candida fermentati, Candida carpophila*, and *Candida xestobii* (Hirayama et al., 2018). Recently, after taxonomic revision, the teleomorph of this yeast was renamed to *Meyerozyma guilliermondii*. However, cases of infections occur due to the anamorph state, still known as *Candida guilliermondii* (Prakash et al., 2017). This complex of species is found colonizing the human skin, the vaginal and oral mucosa. On the other hand, *C. guilliermondii* complex are opportunistic pathogens and can cause several infections. The number of invasive infections associated with yeasts has increased in recent years and *C. guilliermondii* complex accounts for 3.7% of all fungal infections in Latin America. In addition, previous studies have shown reduced susceptibility to azoles and echinocandins among *C. guilliermondii* complex species (Pfaller et al., 2006; Tseng et al., 2016; Marcos-Zambrano et al., 2017; Ahangarkani et al., 2019).

In this study, we report a case of subacute mastitis in a previously healthy postpartum woman caused by *C. guilliermondii*, which was identified by a polyphasic approach.

## Case report

The patient was a Caucasian female, born and resident in the city of Rio de Janeiro, 32 years old, and married. No allergies or comorbidities were reported, denied alcohol and tobacco consumption or continuous use of any medication, and had no history of recent exposure to azoles or methotrexate, which is an antifolate that can induce cross-resistance to azoles (Karuga et al., 2021). In May 2018, at 39 gestational weeks (plus 1 day), according to the patient’s choice, an elective cesarean section was performed, and no complications during childbirth or prenatal care were reported. The newborn was kept in exclusive breastfeeding after birth.

The patient sought medical attention 10 days after delivery complaining of pain in both breasts and presenting bilateral breast fissure. After being instructed on best practices for breastfeeding, a return appointment was scheduled within 7 days, when a significant improvement in the pain and breast fissures was seen. Thirty days after delivery, the infant presented inadequate weight gain, according to the World Health Organization guidelines and growth chart (World Health Organization, 2006), requiring milk complementation using the trans-lactation technique. As the infant evolved effective weight gain, supplementation was gradually decreased. Two months and 15 days after delivery, the patient presented intense burning in both breasts in a symmetrical way, with worsening of symptoms after breastfeeding. No fever or other complaint was reported. The patient reported that there were no changes in hygiene, food, or health-related habits in the postpartum period. Upon clinical examination of the breasts, a mild bilateral areolar hyperemia was observed. The nipples did not present any changes (before or after breastfeeding). Mammary/nipple palpation was painless and did not indicate the presence of any nodule. No signs of candidiasis were seen in the newborn, who was well positioned and attached during breastfeeding.

A milk sample was collected and submitted to culture for bacterial and fungal screening. The patient was prescribed fluconazole 150 mg P.O. once a week for 4 weeks, in addition to the topical application of nystatin in both breasts after breastfeeding for 7 days. The use of oral nystatin in the infant was also recommended, following internal protocols of the institution. It is worth mentioning that fluconazole can pass into breast milk in amounts lower than the dosages used in the treatment of systemic infections and the use of fluconazole during breastfeeding does not pose a risk to infants, according to the American Society of Pediatrics and the Brazilian Ministry of Health (American Academy of Pediatrics, 2001; Brasil, 2010; Kaplan et al., 2015). Although ketoconazole is the drug of choice for mastitis treatment in puerperal women according to the guidelines of the Brazilian Ministry of Health (Brasil, 2002), this medication was not available at the health institution where the patient was assisted; and thus, fluconazole was used following the internal institution’s protocol. The patient was assisted at Instituto Fernandes Figueira (IFF) of Fundação Oswaldo Cruz, which is a reference institution in mother and child health, and is considered by the WHO and the Brazilian Ministry of Health as a “child-friendly hospital.” The study was approved by the institution’s Research Ethics Committee (CAAE 43389321.9.0000.5257). During treatment, the patient continued to breastfeed symmetrically. In addition, the patient was advised to adhere to recommendations related to best hygiene practices (exposing the breasts to air, washing hands before and after breastfeeding, sterilizing objects in contact with the newborn’s mouth). Two weeks after starting the treatment, the patient returned to a new appointment, and showed no improvement in the pain besides presenting a mild desquamation and moderate bilateral areolar hypochromia. The infant had no symptoms or complications. Milk samples, as well as samples of skin areas of both breasts, were collected and submitted to culture. In addition, a second round of fluconazole 150 mg for 7 days was prescribed, according to the internal health unit’s protocol. One week later, the patient returned to the health unit and was fully recovered, with no pain and no areolar lesions Table 1.

**Table 1:** Timetable of patient’s symptoms and treatment.

Patient	Date of medical attendance	Clinical signs and symptoms	Medical recommendation
A female patient, Latin-American, 32 years old, resident in the city of Rio de Janeiro	First attendance – 10 days after delivery	Pain in both breasts and presenting bilateral rashes	Instructed on best practices for breastfeeding
	Return 7 days after	Improvement in the pain and rashes was seen	
	30 days after delivery	The infant presented inadequate weight gain	milk complementation using the trans-lactation technique
	Two months and 15 days after delivery	The patient presented intense burning in both breasts in a symmetrical way, with worsening of symptoms after breastfeeding No fever or other complaint was reported Upon clinical examination of the breasts, a mild bilateral areolar hyperemia was observed The nipples did not present any changes (before or after breastfeeding) Mammary/nipple palpation was painless and did not indicate the presence of any nodule No signs of candidiasis were seen in the baby	The patient was medicated with: fluconazole 150 mg once a week for 4 weeks and topical application of nystatin in both breasts after breastfeeding for 7 days The patient was advised to adhere to recommendations related to best hygiene practices The use of oral nystatin in the infant was also indicated
	Two weeks after starting the treatment	The patient showed no improvement in the pain besides a mild desquamation and moderate bilateral areolar hypochromia	A second round of fluconazole 150 mg for 7 days was prescribed
	Three weeks after starting the treatment	The patient returned to the health unit and was fully recovered, with no pain and no areolar lesions	

**Figure 1:**
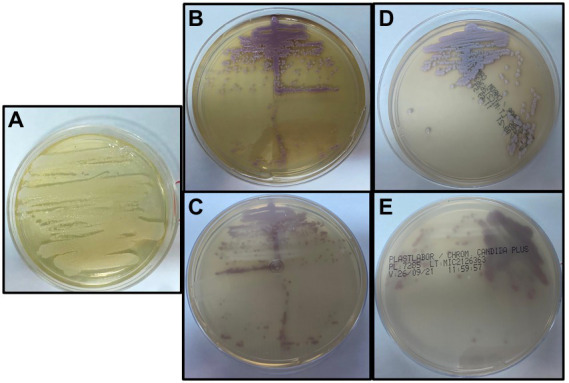
**(A)** Growth on Sabouraud Dextrose Agar after incubation at 30°C for 48 h in aerobic conditions. **(B,C)** Growth on CHROMagar™ Candida after incubation at 35°C for 48 h in aerobic conditions. **(D,E)** Growth on CHROMagar Candida Plus after incubation at 35°C for 48 h in aerobic conditions.

For sample collection, the medical staff used clean medical gloves, and 2 ml of breast milk was collected after discarding the first 1 ml. Samples were transported to the Laboratory of Taxonomy, Biochemistry, and Bioprospection of Fungi at Fundação Oswaldo Cruz, Rio de Janeiro, Brazil within 30 min after sampling and kept at −20°C until analysis. Samples were streaked onto Sabouraud Dextrose Agar (SDA) and incubated at 30°C for 48 h, when the morphological characteristics were then evaluated. No bacterial growth was detected, and fungal colonies with the same morphology and characteristics were observed from all clinical samples (Figure 1A). Growth on SDA was subcultured onto CHROMagar *Candida* (BD Difco) and CHROMagar *Candida* Plus (CHROMagar™) and colonies were interpreted according to the manufacturer’s instructions. Small pink colonies, suggesting *C. guilliermondii*, were observed in both media (Figures 1B–E) from all clinical samples. Biochemical characteristics, examined by conventional methods (Yarrow, 1998; Li et al., 2006), also indicated *C. guilliermondii*. In addition to morphologic and phenotypic tests, the isolate was also identified using molecular tools, including MALDI-TOF MS and sequencing of ITS region.

**Figure 2:**
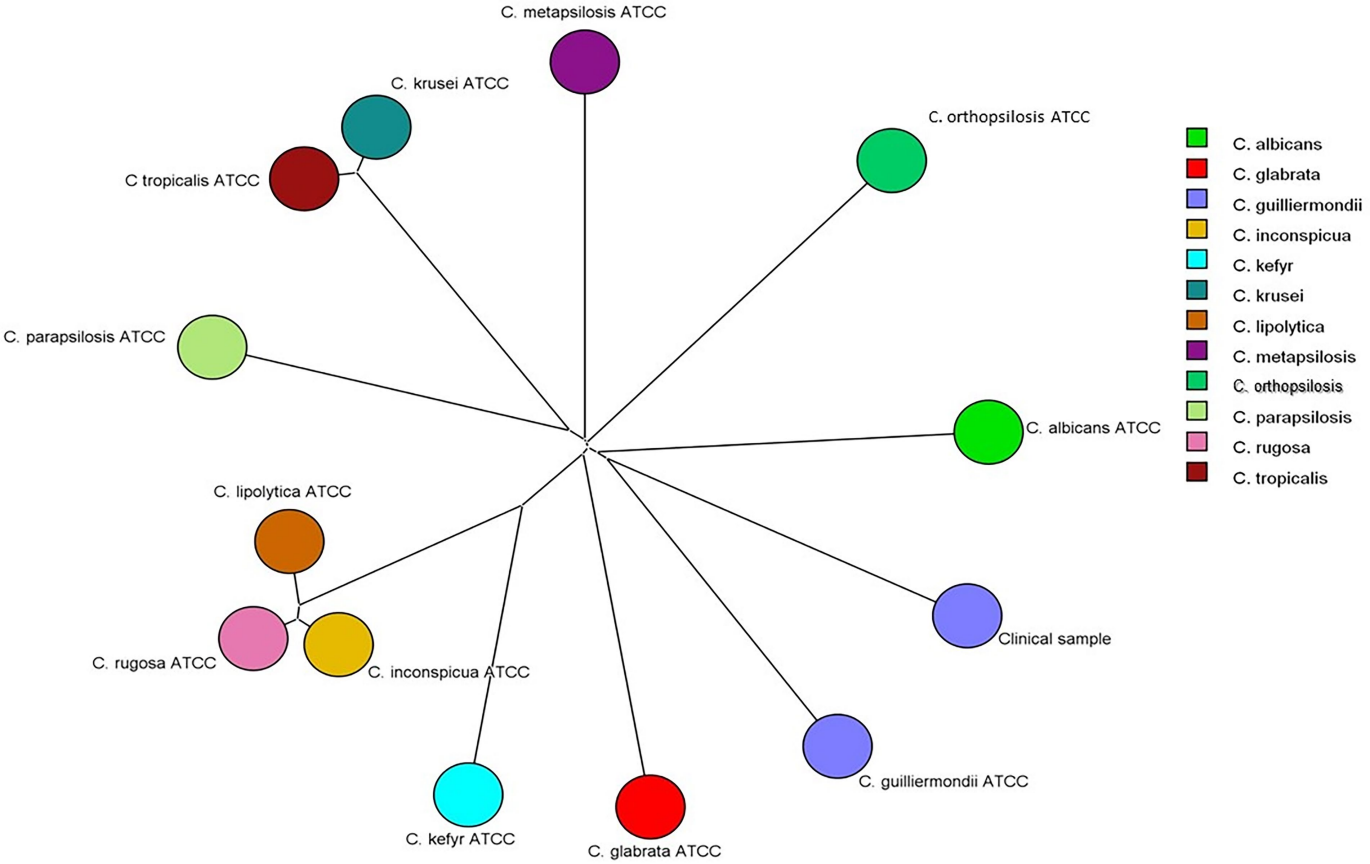
Neighbor-Joining tree based on Pearson correlation constructed with MALDI-TOF MS spectra of the clinical isolate and of reference strains of *Candida albicans* (ATCC 18804), *Candida glabrata* (ATCC 2001), *Candida guilliermondii* (ATCC 7350), *Candida inconspícua* (ATCC 16783), *Candida kefyr* (ATCC 4135), *Candida krusei* (ATCC 6258), *Candida lipolytica* (ATCC 18942), *Candida metapsilosis* (ATCC 96143), *Candida orthopsilosis* (ATCC 96141), *Candida parapsilosis* (ATCC 22019), and *Candida rugosa* (ATCC 10571).

Identification at species level by MALDI-TOF MS was carried out as previously described by Cassagne et al. (2013) and Normand et al. (2019); Matos et al. (2020) with few modifications. Briefly, 10^6^ yeast cells were transferred from the culture plate (c.a. 1g) to a 500 μl tube containing 20 μl of 70% formic acid in water (v/v). The supernatant of each sample (1 μl) was transferred to a paraffin film surface and 10 μl of acetonitrile was added. The sample (1 μl) was spotted onto the MALDI-TOF MS stainless plate (Bruker Daltonics, Germany) and covered with 1 μl matrix solution α-cyano-4-hydroxycinnamic acid (CHCA, Fluka, Buchs, Switzerland). Each sample was analyzed in triplicate. The sample was air-dried at room temperature previous to the spectra acquisition. Using the Bruker database, it was possible to identify the isolate at species level (score of 1.72) as *C. guilliermondii*. The spectra generated were exported to BioNumerics software v8.1 (Applied Maths) with which a Neighbor-Joining tree was created using MALDI-TOF spectra generated from reference strains representing major *Candida* species. The clinical isolate was clustered together with *C. guilliermondii* reference strain (Figure 2) confirming the automated identification by MALDI-TOF MS.

**Figure 3:**
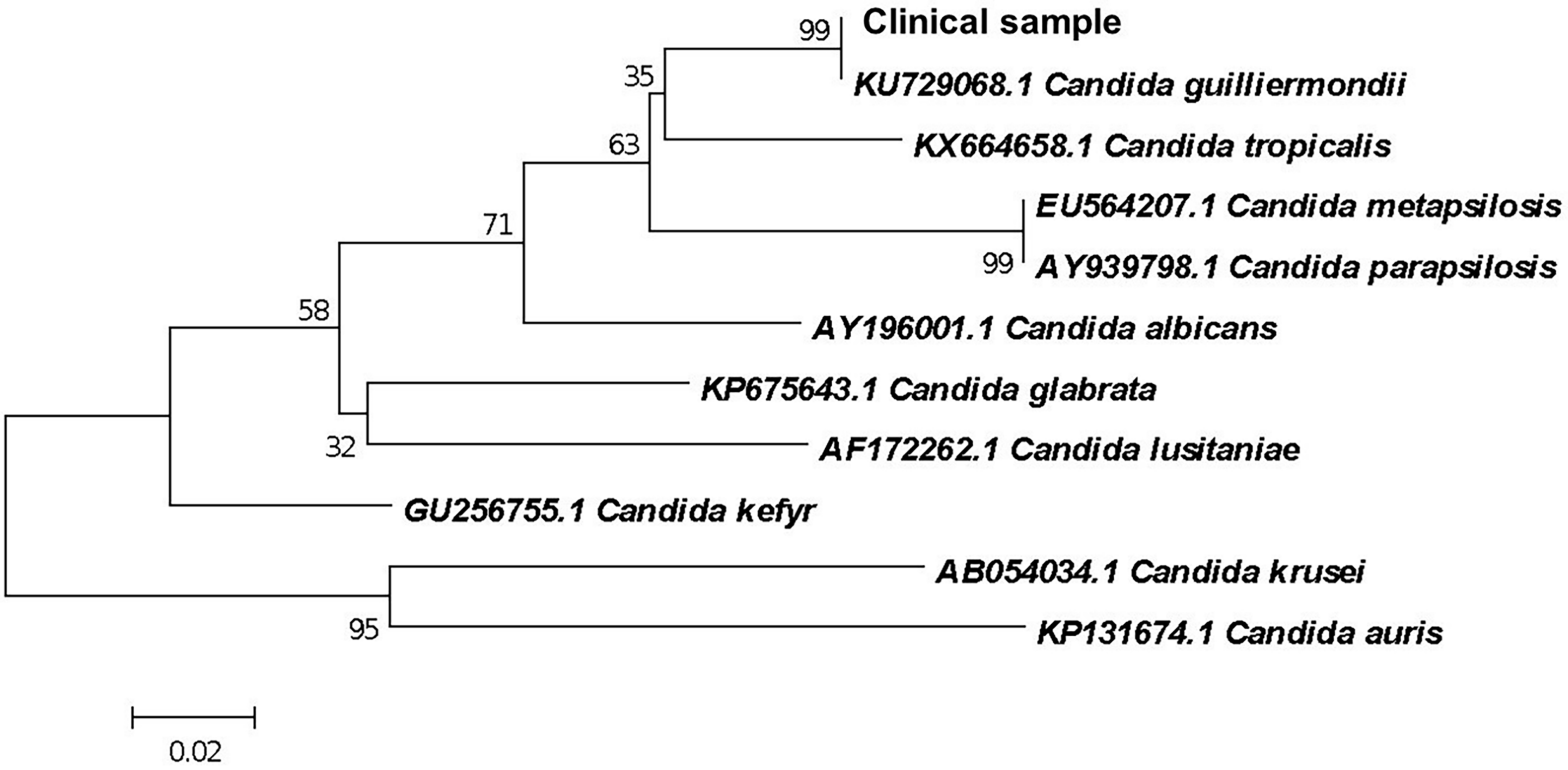
Phylogenetic relationship between the clinical isolate and reference strains of Candida species inferred from ITS sequences by Neighbor-Joining method (Saitou and Nei, 1987). The optimal tree is shown. The percentages of replicate trees in which the associated taxa clustered together in the bootstrap test (1,000 replicates) are shown next to the branches (Felsenstein, 1985). Branches corresponding to partitions reproduced in less than 50% bootstrap replicates are collapsed. The evolutionary distances were computed using the Maximum Composite Likelihood method (Tamura et al., 2004) and are in the units of the number of base substitutions per site. This analysis involved 11 nucleotide sequences. There were a total of 78 positions in the final dataset. Evolutionary analyses were conducted in MEGA X (Tamura et al., 2007).

From colonies grown in SDA culture, genomic DNA was extracted using the Gentra^®^ Puregene^®^ Yeast and G+ Bacteria kit (Qiagen^®^, Maryland, United States) according to the manufacturer’s recommendations. The amplification of the ITS1-5.8S-ITS2 region of ribosomal DNA was performed in a final volume of 50 μl containing 100 ng of DNA and 25 ng/μL of each primer (Invitrogen^TM^ Brazil), ITS1 (5′ TCCGTAGGTGAACCTGCGG 3′) and ITS4 (5′TCCTCCGCTTATTGATATGC 3′) (Lindsley et al., 2001). PCR was performed in an Applied Biosystems thermocycler (model Veriti) with annealing temperature of 50°C. The amplified product was purified with the QIAquick^®^ PCR Purification Kit (QIAGEN^®^) and sequenced using the ABI-3730 sequencer (Applied Biosystems). The sequences were edited using CodonCode Aligner (Genes Code Corporation, Ann Arbor, United States), and phylogenetic analysis was performed using Blast software for comparison with the sequences deposited in the NCBI/GenBank database. An identity of 100% of the clinical isolate when compared to the reference sequence of *C. guilliermondii* (ATCC6260) KU729068.1 deposited at NCBI/GenBank was observed (Figure 3).

## Discussion

A previous study reported the detection of *Candida albicans* from breast milk samples in 23% of the women with symptoms of deep breast pain with or without nipple/areola symptoms (Kaski and Kvist, 2018). Mutschlechner et al. (2016) in Austria reported C*. guilliermondii* in milk sample recovered from a woman with breast infection. Nevertheless, correlation of *Candida* infections with problems in lactation is generally made only by clinical assessment of patients, without carrying out microbiological analysis (Brent, 2001). The identification of *Candida* in maternal infections is of great importance since vertical transmission to the neonate is the main form of neonatal colonization, followed by nosocomial horizontal transmission. Neonatal candidemia is an important cause of mortality, especially in intensive care units, being associated with up to 30% of neonatal deaths (Waggoner-Fountain et al., 1996; Tiraboschi et al., 2010; Lamba et al., 2021).

In this study, MALDI-TOF MS showed to be a fast and reliable tool for identifying the fungal species associated with the infection, as the results were in accordance with morphological/phenotypic approach and genetic sequencing. Indeed, MALDI-TOF has been highlighted by several other studies as a useful technique to identify fungal species at the genus and species level (Santos et al., 2011; Oliveira et al., 2015; Marín et al., 2017; Valero et al., 2018; Kal Çakmaklıoğulları et al., 2019).

There is an increasing body of evidence that certain fungi may compose the human milk microbiome (Boix-Amorós et al., 2017; Moossavi et al., 2020). However, since identification at species level of these commensal fungi is still rarely performed, it is unknown whether *C. guilliermondii* could be part of human milk microbiota, and, thus, be considered a potential contaminant of milk samples. In the present study, the absence of bacterial growth and the finding of a single colony type from all clinical samples strongly suggests that this single isolate, which turned out to be identified as *C. guilliermondii*, was associated with the mastitis case reported. The use of fluconazole to treat the patient followed the internal protocol of the healthcare institution assisting the patient, as well as it falls within national guidelines (Brasil, 2010). Although fluconazole is not the first choice for mastitis treatment, especially due to possible secretion in human milk and association with higher risks of resistance development, it still represents an alternative choice for lactating women as it is safe for infants. Moreover, the fact that the patient completely recovered from the subacute mastitis after the second round of fluconazole suggests that the *C. guilliermondii* strain associated with this case report was susceptible to fluconazole, even though the antimicrobial susceptibility testing was not performed.

Despite the increasing number of infections caused by non-*albicans Candida*, such as *C. guilliermondii*, studies and efforts to identify the etiological agent of fungal mastitis are still lacking (Amir et al., 1996). The rapid and correct diagnosis in such cases is essential to avoid mistreatment and to ensure the maintenance of breastfeeding, which has a huge impact in both the mother and the newborn. Cases similar to the one reported here could benefit from a fast and accurate microbiological identification approach such as MALDI-TOF MS, ensuring a more effective therapeutic option and, thus, a better outcome for mother and child.

## Data availability statement

The original contributions presented in the study are included in the article, further inquiries can be directed to the corresponding author.

## Ethics statement

The study was approved by the institution’s Research Ethics Committee under the number CAAE 43389321.9.0000.5257.

## Author contributions

TNP and AK: methodology, investigation, formal analysis, and writing – original draft. GC: supervision, visualization, and writing – review and editing. TCP: conceptualization and writing – review and editing. LO: visualization and writing – review and editing. MO: conceptualization, resources, supervision, project administration, funding acquisition, and writing – review and editing. All authors contributed to the article and approved the submitted version.

## Funding

This study was supported by CAPES (Coordenação de Aperfeiçoamento de Pessoal de Nível Superior – D.C.M fellowship 88882.317297/2019–01 and Finance Code 001), and Fundaçao Carlos Chagas Filho de Amparo à Pesquisa do Estado do Rio de Janeiro (MO; FAPERJ – Grants: JCNE E-26/203.301/2017; JCNE E-26/201.433/2021; MO and TCP Grant: E-26/010.002141/2019).

## Conflict of interest

The authors declare that the research was conducted in the absence of any commercial or financial relationships that could be construed as a potential conflict of interest.

## Publisher’s note

All claims expressed in this article are solely those of the authors and do not necessarily represent those of their affiliated organizations, or those of the publisher, the editors and the reviewers. Any product that may be evaluated in this article, or claim that may be made by its manufacturer, is not guaranteed or endorsed by the publisher.
